# Supplementary material for: Role of Whole Grain Consumption in Glycaemic Control of Diabetic Patients: A Systematic Review and Meta-Analysis of Randomized Controlled Trials
Source: Nutrients. 2021 Dec 27;14(1):109. doi: 10.3390/nu14010109 (PMC8746707; doi:10.3390/nu14010109)
Supplement: Supplementary file 1 [file nutrients-14-00109-s001.zip › nutrients-1509860-supplementary.pdf]

Supplemental files - Table S1. The detailed search strategies and results in the databases.

Database	Search strategies	Results
Web of science	(((((((TS=(whole grains)) OR TS=(whole-grains)) OR TS=(grains)) OR TS=(cereals)) OR TS=(whole wheat)) OR TS=(barely)) OR TS=(oat)) AND TS=(diabetes mellitus)	3353 items
Pubmed	("whole grains"[MeSH Terms] OR ("whole"[All Fields] AND "grains"[All Fields]) OR "whole grains"[All Fields] OR ("whole grains"[MeSH Terms] OR ("whole"[All Fields] AND "grains"[All Fields]) OR "whole grains"[All Fields]) OR ("cyprinidae"[MeSH Terms] OR "cyprinidae"[All Fields] OR "graining"[All Fields] OR "edible grain"[MeSH Terms] OR ("edible"[All Fields] AND "grain"[All Fields]) OR "edible grain"[All Fields] OR "grain"[All Fields] OR "grain s"[All Fields] OR "grained"[All Fields] OR "grains"[All Fields]) OR ("cereale"[All Fields] OR "edible grain"[MeSH Terms] OR ("edible"[All Fields] AND "grain"[All Fields]) OR "edible grain"[All Fields] OR "cereal"[All Fields] OR "cereals"[All Fields]) OR (("whole"[All Fields] OR "wholeness"[All Fields] OR "wholes"[All Fields]) AND ("triticum"[MeSH Terms] OR "triticum"[All Fields] OR "wheat"[All Fields] OR "wheat s"[All Fields] OR "wheats"[All Fields])) OR "barely"[All Fields] OR "oat"[All Fields]) AND ("diabetes mellitus"[MeSH Terms] OR ("diabetes"[All Fields] AND "mellitus"[All Fields]) OR "diabetes mellitus"[All Fields])	1466 items
Scopus	(TITLE-ABS-KEY (diabetes AND mellitus) AND TITLE-ABS-KEY (whole AND grains) OR TITLE-ABS-KEY (whole-grains) OR TITLE-ABS-KEY (grains) OR TITLE-ABS-KEY (cereals) OR TITLE-ABS-KEY (whole AND wheat) OR TITLE-ABS-KEY (barely) OR TITLE-ABS-KEY (oat))	2376 items
Cochrane library	whole grains or whole-grains or grains or cereals or whole wheat or barely or oat in Title Abstract Keyword AND diabetes mellitus in Title Abstract Keyword	623 items

Supplemental Files-Table S2. Quality assessment of included studies based on the Cochrane guidelines.

Study	random sequence generation	allocation concealment	blinding of participants and personnel	blinding of outcome assessment	incomplete outcome data	selective reporting	other sources of bias
Elbalshy et al. 2021	L	L	L	L	L	L	L
Barati et al. 2021	L	H	U	L	L	L	L
Pavithran et al. 2020	U	U	U	L	L	L	L
Malin et al. 2019	L	L	L	L	L	L	L
Delgado et al. 2019	L	H	L	U	L	L	L
Liu et al. 2018	U	L	H	L	L	L	L
Kondo et al. 2017	L	L	H	L	L	L	L
Li et al. 2016	L	L	U	L	L	L	L
Islam et al. 2015	U	U	U	L	L	L	L
Ma et al. 2013	L	L	L	L	L	L	L
Lankinen et al. 2011	U	U	H	L	L	L	L
Hsu et al.2008	U	L	L	L	L	L	L
Rave et al. 2007	U	L	L	L	L	L	L
Rendell et al.2005	U	L	L	L	L	L	L
Jenkins et al. 2002	U	L	L	L	L	L	L
Pick et al. 1996	U	U	U	L	L	L	L

L: low risk, U: unclear, H: high risk.

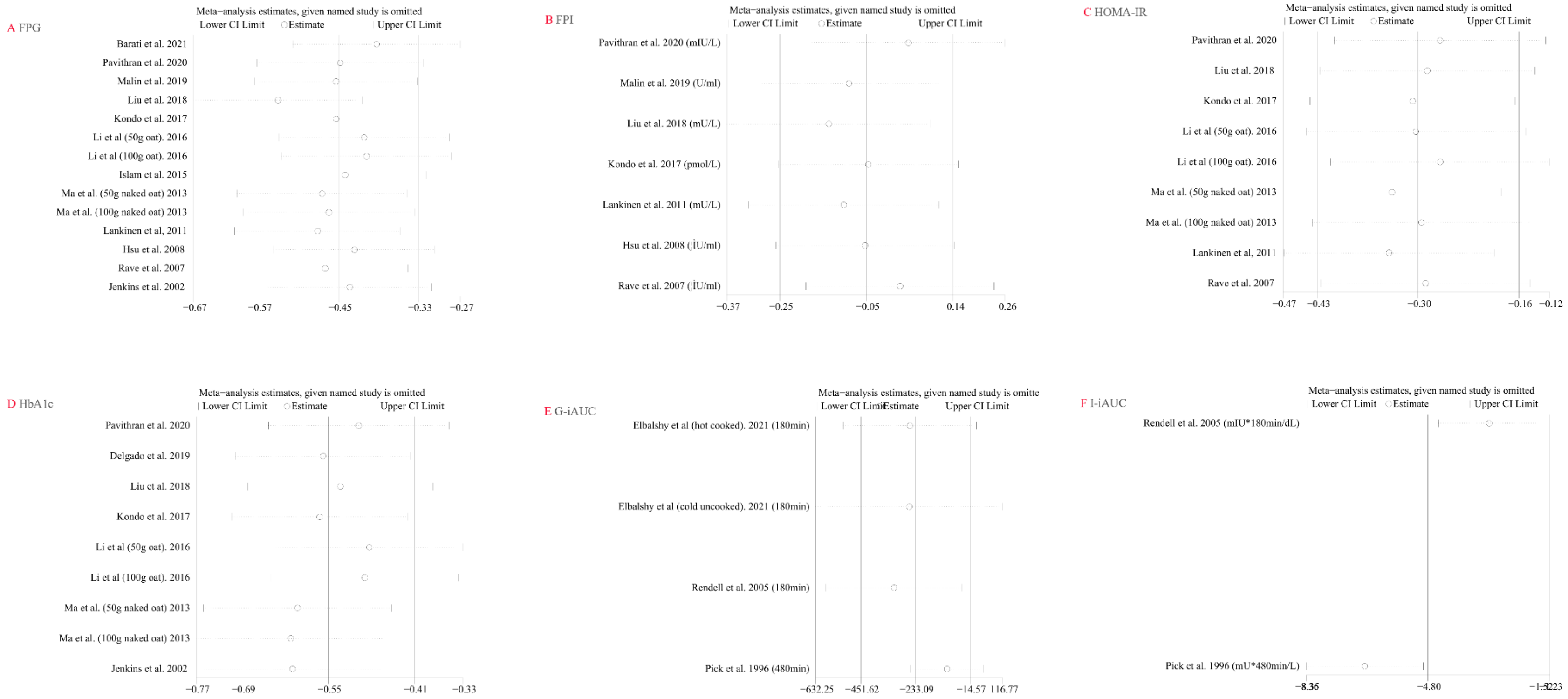

Supplemental files - Figure S1. Result of sensitivity analysis.

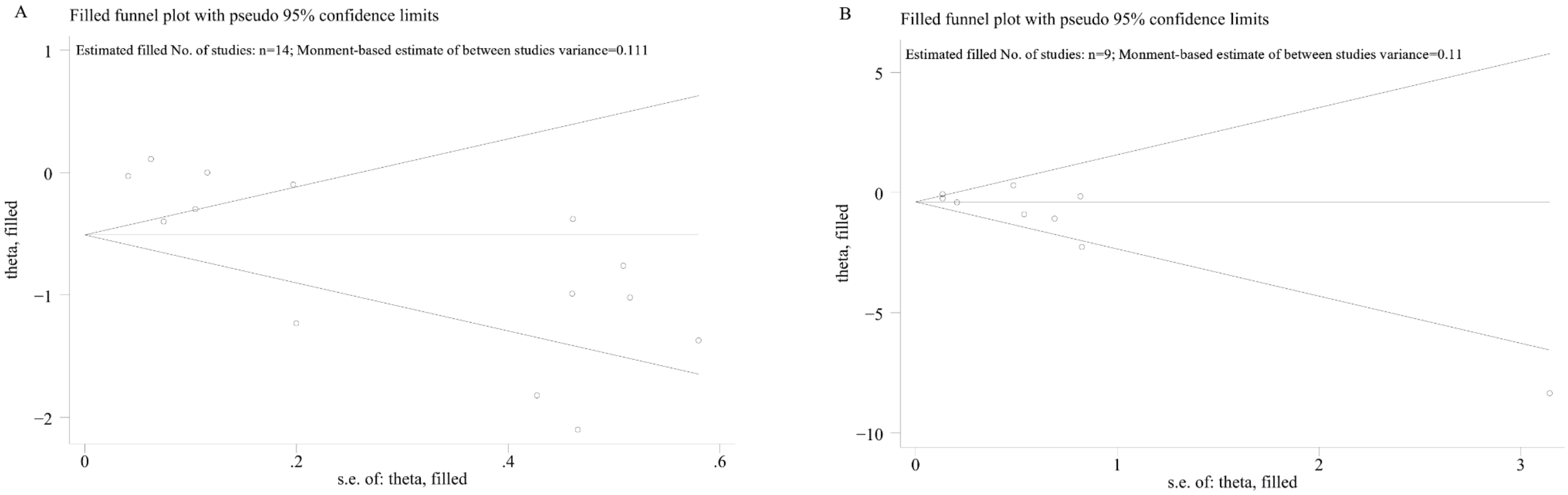

Supplemental files - Figure S2. Result of “trim and fill” analysis (A: FPG, B:HOMA-IR).
